# Sex- and Diabetes-Dependent Perioperative Model for End-Stage Liver Disease Trajectories Identify Distinct Hepatorenal Stress Phenotypes After Surgical Coronary Revascularization

**DOI:** 10.3390/jcm15082906

**Published:** 2026-04-11

**Authors:** Tomasz Urbanowicz, Monika Bajsert, Ewelina Grywalska, Krzysztof J. Filipiak, Beata Krasińska, Paulina Mertowska, Monika Kowalczyk, Sebastian Mertowski, Zuzanna Marcinkowska, Mansur Rahnama, Oksana Wiśniewska, Julia Gierszewska, Anna Olasińska-Wiśniewska, Ewelina Swora-Cwynar, Krzysztof Bartuś, Zbigniew Krasiński, Assad Haneya, Marek Jemielity

**Affiliations:** 1 Cardiac Surgery and Transplantology Department, Poznan University of Medical Sciences, ½ Długa Street, 61-848 Poznan, Poland; 2 Cardiac Surgery Students’ Research Group, Poznan University of Medical Sciences, 10 Fredry Street, 61-701 Poznan, Poland; 3 Department of Experimental Immunology, Medical University of Lublin, 6 Chodźki Street, 20-093 Lublin, Poland; 4 The Centre of Postgraduate Medical Education, 99/103 Marymoncka Street, 01-813 Warsaw, Poland; 5 Department of Hypertensiology, Angiology, and Internal Medicine, Poznan University of Medical Sciences, ½ Długa Street, 61-848 Poznan, Poland; 6 Department of Dental Surgery, Medical University of Lublin, 6 Chodźki Street, 20-093 Lublin, Poland; 7 Department of Gastroenterology, Diabetes and Internal Diseases, Poznan University of Medical Sciences, 49 Przybyszewskiego Street, 61-701 Poznan, Poland; 8 Department of Cardiovascular Surgery and Transplantology, Jagiellonian University Medical College, 80 Pradnicka Street, 31-202 Cracow, Poland; 9 Department of Vascular, Endovascular Surgery, Angiology and Phlebology, Poznan University of Medical Sciences, ½ Dluga Street, 61-848 Poznan, Poland; 10 Department of Cardiothoracic Surgery, Heart Centre Trier, Barmherzigen Brueder Hospital, 54292 Trier, Germany

**Keywords:** MELD score, off-pump coronary artery bypass grafting, perioperative risk stratification, sex differences, diabetes mellitus, hepatorenal response

## Abstract

**Background/Objectives**: Perioperative risk stratification in cardiac surgery is based mainly on static preoperative variables and therefore does not fully capture dynamic multiorgan responses to surgical stress. The Model for End-Stage Liver Disease (MELD) score, which integrates bilirubin, creatinine, and the international normalized ratio (INR), reflects hepatorenal function, but its perioperative dynamics remain insufficiently explored. This study aimed to characterize perioperative MELD trajectories in patients undergoing off-pump coronary artery bypass grafting (OPCAB) and to assess the influence of sex and diabetes mellitus on these changes and their clinical relevance. **Methods**: This retrospective observational study included 111 patients undergoing elective OPCAB. MELD scores were assessed preoperatively (MELD_0_), on postoperative day 1 (MELD_1_), and on day 6 (MELD_6_). Dynamic indices of MELD change were calculated, including the early postoperative increase (ΔMELD_01_). The effects of sex and diabetes mellitus on MELD trajectories were analyzed using multivariable linear regression and generalized estimating equations. A high-surge phenotype was defined as the upper quartile of ΔMELD_01_. **Results**: MELD increased significantly on postoperative day 1 and partially recovered by day 6 (*p* < 0.001). Female sex was independently associated with lower postoperative MELD values (β = −2.54, *p* < 0.001) and a smaller ΔMELD_01_, whereas diabetes mellitus was associated with a reduced MELD rise (β = −1.07, *p* = 0.028). Patients with a high-surge MELD phenotype had significantly longer hospitalization than those with a lower MELD response (12.8 ± 2.1 vs. 9.2 ± 1.2 days, *p* < 0.001). **Conclusions**: Perioperative MELD trajectories reflect a dynamic hepatorenal stress response after OPCAB and may improve identification of clinically relevant physiological vulnerability.

## 1. Introduction

Perioperative risk stratification in cardiac surgery has traditionally relied on composite scoring systems based predominantly on static preoperative variables. Although these models remain clinically useful, they do not fully capture the dynamic and interconnected responses of multiple organ systems to surgical stress. Among these, hepatic dysfunction remains relatively underrecognized, despite its central role in systemic inflammation, coagulation, metabolic regulation, and maintenance of homeostasis. The Model for End-Stage Liver Disease (MELD) score, originally developed to predict survival in patients with advanced liver disease, incorporates serum bilirubin, creatinine, and the international normalized ratio (INR), thereby providing an integrated reflection of hepatorenal functional status [1].

Increasing evidence suggests that MELD may also serve as a meaningful predictor of postoperative morbidity and mortality in patients undergoing cardiac surgery [2]. However, prior investigations have focused primarily on baseline MELD values, implicitly treating hepatic dysfunction as a static preoperative condition. In contrast, cardiac surgery induces rapid and profound physiological perturbations, including ischemia–reperfusion injury, systemic inflammatory activation, oxidative stress, and microcirculatory dysfunction, all of which may result in acute but potentially reversible derangements in hepatic and renal function [3]. Such dynamic postoperative changes are not adequately captured by conventional risk models.

Previous studies [4,5,6,7] have highlighted the relevance of perioperative biomarker dynamics in cardiac surgery, particularly with respect to inflammatory and metabolic responses to operative stress. In this context, temporal changes in biochemical indices may more accurately reflect acute physiological perturbation than isolated preoperative measurements, supporting the concept of dynamic risk assessment in the perioperative setting. 

Although MELD was originally designed for patients with advanced liver disease, its individual components—bilirubin, creatinine, and INR—represent key dimensions of hepatorenal and coagulation homeostasis that are highly relevant in the perioperative period. Accordingly, perioperative fluctuations in MELD may capture an integrated response to inflammation, hypoperfusion, and coagulation disturbances that is not represented in traditional cardiac surgical risk scores.

In parallel, sex and metabolic status are increasingly recognized as important modifiers of perioperative physiology [8,9,10,11]. Biological sex influences inflammatory signaling, endothelial function, vascular reactivity, and mitochondrial resilience, whereas diabetes mellitus is associated with chronic metabolic adaptation, altered substrate utilization, endothelial dysfunction, and potentially modified responses to acute stress [12,13]. Whether these factors influence perioperative MELD trajectories, however, has not been systematically investigated.

We hypothesized that perioperative changes in MELD, rather than baseline values alone, reflect distinct patterns of physiological response following surgical coronary revascularization and that these trajectories are modulated by sex and diabetic status. The present study sought to characterize perioperative MELD kinetics, determine the independent effects of sex and diabetes mellitus on MELD trajectories, and examine whether a high-risk MELD surge phenotype is associated with exaggerated hepatorenal dysfunction.

## 2. Materials and Methods

### 2.1. Design and the Study Setting

Consecutive adult patients who underwent elective off-pump coronary artery bypass grafting (OPCAB) at a tertiary care academic center in 2024 were included in the observational study. All the data was collected retrospectively. The study was designed to evaluate perioperative changes in the Model for End-Stage Liver Disease (MELD) score as a dynamic marker of hepatorenal stress response following surgical myocardial revascularization. Particular attention was paid to temporal MELD trajectories and to the potential modifying effects of biological sex and diabetes mellitus.

A total of 111 patients were included in the final analysis, comprising 87 men and 24 women, with a median age of 68 years (interquartile range [IQR], 63–72 years). The study population consisted exclusively of patients undergoing elective OPCAB, which allowed the establishment of a relatively homogeneous cohort with respect to surgical technique and perioperative exposure.

Eligibility required the availability of complete perioperative laboratory data necessary for MELD calculation at all predefined time points. To reduce confounding by pre-existing organ dysfunction, patients with clinically significant chronic liver or kidney disease were excluded. Chronic kidney disease was defined as stage ≥3 according to KDIGO classification (estimated glomerular filtration rate < 60 mL/min/1.73 m^2^). Chronic liver disease was defined as documented cirrhosis, chronic viral hepatitis, or other clinically relevant hepatic dysfunction associated with persistent biochemical abnormalities (e.g., elevated bilirubin or INR). Additional exclusion criteria included systemic inflammatory disorders (e.g., rheumatoid arthritis, systemic lupus erythematosus), as well as systemic diseases potentially affecting hepatic or renal function, such as active malignancy, severe chronic infections, or advanced heart failure with end-organ dysfunction. Patients with perioperative complications associated with postoperative low cardiac output syndrome were also excluded to avoid confounding effects of hemodynamic instability on postoperative biochemical parameters.

These criteria were applied to improve cohort homogeneity and to ensure that observed MELD changes primarily reflected perioperative physiological stress rather than overt baseline organ failure or major postoperative instability.

Patients reporting restrictive diets or food allergies were also excluded from the analysis in order to minimize potential metabolic and nutritional confounders that might influence perioperative biochemical parameters relevant to MELD calculation and interpretation. Before statistical analysis, the dataset underwent internal consistency verification to confirm completeness and coherence of the variables used for MELD calculation and subsequent modeling. Only cases with complete data available at all predefined perioperative time points were retained in the final analytical cohort.

The study was conducted in accordance with the ethical principles of the Declaration of Helsinki. Ethical approval was obtained from the Institutional Ethics Committee of Poznan University of Medical Sciences, Poznan, Poland (protocol no. 612/25; approved on 11 September 2025). Prior to the analysis, all participants completed a written informed consent form.

### 2.2. Data Collection and Clinical Definitions

Clinical, demographic, and laboratory data were obtained from a prospectively maintained institutional database. The extracted dataset included baseline demographic characteristics, cardiovascular risk factors, comorbidities, perioperative variables, and serial biochemical measurements collected as part of routine perioperative care.

Total hospitalization time was defined as the interval from admission to discharge from the cardiac surgery department. Postoperative hospitalization time included both intensive care unit stay and the subsequent period of postoperative recovery and rehabilitation within the cardiac surgery department.

For baseline characterization, dyslipidemia was defined as a documented history of hyperlipidemia and/or current lipid-lowering therapy, irrespective of cholesterol levels measured at admission. Consequently, baseline lipid values reflected treated rather than untreated metabolic status in a substantial proportion of patients.

Diabetes mellitus was defined on the basis of a documented diagnosis and/or ongoing antidiabetic treatment at the time of hospitalization. Sex was analyzed as a biological variable and categorized as female or male.

Postoperative venous thromboembolism prophylaxis was routinely administered with low-molecular-weight heparin (enoxaparin) according to institutional protocols (20 to 40 mg daily, adjusted by GFR).

### 2.3. MELD Assessment and Perioperative Time Points

The MELD score was calculated using the standard formula based on serum bilirubin, creatinine, and the international normalized ratio (INR), thus integrating hepatic, renal, and coagulation-related parameters into a single composite index. In the present study, MELD was used not as a marker of chronic liver disease severity, but as an integrated indicator of perioperative physiological disturbances involving the hepatorenal axis and hemostatic balance.

Laboratory measurements required for MELD calculation were collected at three predefined perioperative time points: before surgery as the preoperative baseline (MELD_0_), on postoperative day 1 as a marker of the early acute response to surgical stress (MELD_1_), and on postoperative day 6 as an indicator of short-term recovery or persistence of postoperative biochemical disturbance (MELD_6_). This temporal framework was selected to capture both the immediate postoperative rise and the early recovery profile.

### 2.4. Derived MELD Dynamics and Phenotype Definition

To characterize perioperative MELD kinetics, several derived indices were calculated. The early postoperative MELD rise was defined as the difference between MELD on postoperative day 1 and the preoperative baseline (ΔMELD_01_ = MELD_1_ − MELD_0_). This parameter was considered the principal marker of the acute perioperative hepatorenal response. The late MELD change was defined as the difference between MELD on postoperative day 6 and baseline (ΔMELD_06_ = MELD_6_ − MELD_0_), reflecting the net deviation from preoperative status after the early postoperative phase. In addition, a recovery index was calculated as the difference between MELD on postoperative day 6 and MELD on postoperative day 1 (MELD_6_ − MELD_1_), allowing assessment of the extent of biochemical normalization after the initial postoperative increase.

Although the recovery index (MELD_6_ − MELD_1_) was calculated, it was used for exploratory assessment of recovery dynamics and was not included in the primary statistical comparisons or tabulated results, as it did not provide additional discriminatory value beyond ΔMELD_06_.

To identify patients with an exaggerated perioperative response, a high-surge phenotype was defined a priori as the upper quartile of the ΔMELD_01_ distribution. This categorization was intended to distinguish individuals with the most pronounced acute hepatorenal perturbation from the remainder of the cohort.

### 2.5. Exposure Variables and Covariates

The principal exposure variables of interest were biological sex and diabetes mellitus. These factors were selected based on their recognized roles in modulating inflammatory, metabolic, endothelial, and organ-specific responses to acute surgical stress.

In multivariable models, additional covariates were included to account for clinically relevant baseline and treatment-related influences on MELD trajectories. These covariates comprised age, baseline MELD score, and the use of sodium-glucose cotransporter 2 (SGLT2) inhibitors. Baseline MELD was included to account for interindividual differences in preoperative hepatorenal status, whereas age and SGLT2 inhibitor use were considered potential modifiers of perioperative metabolic and renal adaptation.

### 2.6. Statistical Analysis

The medians with interquartile ranges describe continuous variables, reflecting the non-normal distribution of the data, whereas categorical variables are reported as absolute counts and percentages. Comparisons between groups for continuous variables were performed using the Mann–Whitney U test, while categorical variables were compared using Fisher’s exact test.

To examine factors independently associated with postoperative MELD response, multivariable linear regression models were constructed using MELD on postoperative day 1 (MELD_1_) and the early postoperative MELD increase (ΔMELD_01_) as dependent variables. These models included sex, diabetes mellitus, baseline MELD, age, and SGLT2 inhibitor use as covariates.

Because MELD was repeatedly assessed over time within the same individuals, longitudinal perioperative MELD trajectories were also analyzed using generalized estimating equations (GEE) with an exchangeable correlation structure. This approach enabled modeling of within-subject dependence and provided estimates of MELD changes across the perioperative period in relation to the investigated modifiers.

To identify independent predictors of the high-surge phenotype, logistic regression analysis was performed. This model was intended to determine whether sex, diabetes mellitus, and selected clinical covariates were associated with an increased or decreased likelihood of exhibiting an exaggerated early postoperative MELD rise.

Given the exploratory nature of multiple pairwise comparisons, *p*-values were not adjusted for multiple testing. All statistical tests were two-sided, and *p*-values < 0.05 were considered statistically significant. Statistical analyses were performed using JASP software, version 0.14.1 (University of Amsterdam, Amsterdam, The Netherlands).

### 2.7. GenAI Statement

GenAI was used to generate the graphical abstract. 

## 3. Results

### 3.1. Baseline Characteristics of the Study Cohort

The study cohort comprised 111 patients undergoing elective off-pump coronary artery bypass grafting, including 87 men (78.4%) and 24 women, with a median age of 68 years (Q1–Q3, 63–72 years). No perioperative deaths were observed. The mean number of distal anastomoses was 2.1 ± 0.6 per patient. The mean overall hospitalization time was 11.0 ± 3.6 days, whereas the mean postoperative hospitalization time was 7.7 ± 1.8 days.

For the primary analyses, patients were stratified by sex and, subsequently, by diabetes mellitus status (Table 1). Women were older than men and had lower body mass index values. Dyslipidemia was more frequent in men, whereas peripheral artery disease was more common in women. Sex-related differences were also observed in selected echocardiographic parameters, including right ventricular diameter and interventricular septal thickness, both preoperatively and postoperatively. In contrast, no significant sex-related differences were identified with respect to hypertension, diabetes prevalence, smoking status, prior stroke, the extent of coronary artery disease, or left ventricular ejection fraction. Likewise, comparisons by diabetes status did not reveal major differences in baseline demographic, coronary, or echocardiographic characteristics, indicating that the principal analytical subgroups were broadly comparable clinically.

### 3.2. Perioperative MELD Dynamics Across Sex–Diabetes Phenotypes

Perioperative laboratory parameters were analyzed across four phenotype-defined subgroups: men without diabetes, men with diabetes, women without diabetes, and women with diabetes. Across the whole cohort, MELD increased substantially on postoperative day 1 and declined toward baseline by postoperative day 6, consistent with an acute but consistent with an acute perioperative hepatorenal stress response characterized by substantial early deterioration followed by incomplete normalization by postoperative day 6. Although creatinine and bilirubin tended to return toward baseline values, INR remained mildly elevated, suggesting that recovery of coagulation homeostasis may lag behind renal and hepatic biochemical parameters (Table 2).

Women exhibited significantly lower MELD values than men throughout the perioperative period. At baseline, median MELD was lower in women than in men (5.08 [4.40–5.83] vs. 5.95 [5.32–6.92], *p* = 0.002). This difference became more pronounced after surgery. On postoperative day 1, women had significantly lower MELD values than men (13.29 [11.56–14.81] vs. 15.94 [14.89–17.58], *p* < 0.001). The early postoperative MELD increase was likewise attenuated in women compared with men (ΔMELD_01_: 7.82 [6.86–9.04] vs. 10.10 [8.88–11.03], *p* < 0.001). These differences were accompanied by lower postoperative bilirubin, creatinine, and INR values, indicating a less pronounced integrated hepatorenal and coagulation response to surgical stress.

Baseline MELD did not differ significantly between patients with and without diabetes mellitus. However, diabetes mellitus was associated with a significantly attenuated early postoperative MELD rise (8.88 [7.81–10.19] vs. 10.10 [8.92–11.18], *p* = 0.020). This effect appeared to be driven primarily by lower postoperative bilirubin and INR values, whereas creatinine changes were less uniform across subgroups.

The recovery index (MELD_6_–MELD_1_) was negative across all subgroups, confirming a consistent decline in MELD following the early postoperative peak, with no significant differences between sex- or diabetes-defined phenotypes.

**Figure 1:**
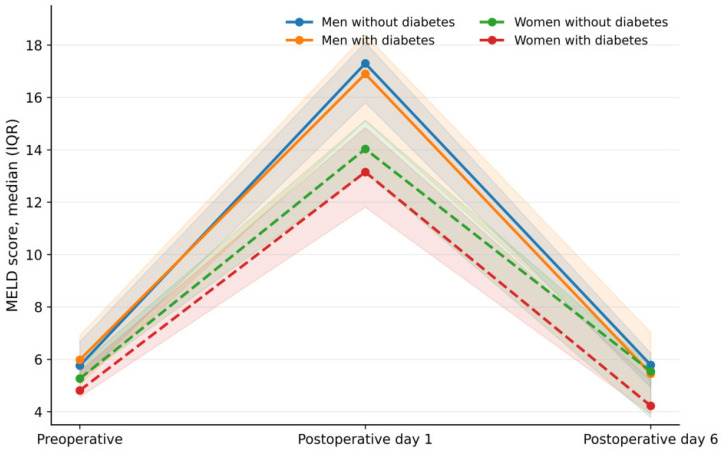
MELD trajectories across the four subgroups.

**Figure 2:**
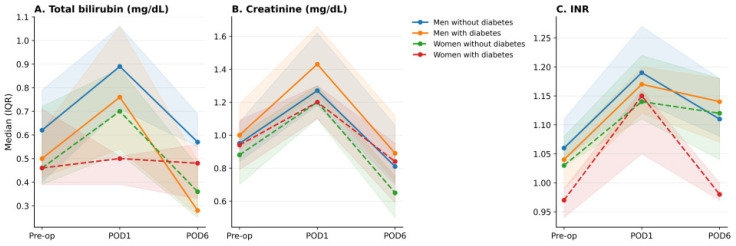
Component-level perioperative changes in bilirubin (**A**), creatinine (**B**), and INR (**C**).

**Figure 3:**
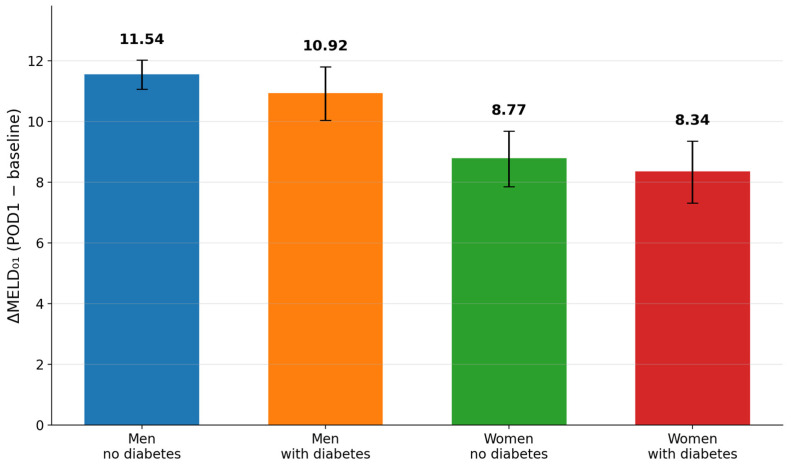
Overall early hepatorenal stress responses across phenotype-defined groups.

Stratification by both sex and diabetes status revealed a graded pattern of physiological responses. Men without diabetes demonstrated the highest postoperative MELD values and the greatest early MELD increase, followed by men with diabetes and women without diabetes, whereas women with diabetes exhibited the lowest MELD response. Thus, perioperative MELD trajectories showed a stepwise attenuation across sex–diabetes phenotypes. Differences in integrated MELD trajectories across the four subgroups are illustrated in Figure 1, component-level perioperative changes in bilirubin, creatinine, and INR are shown in Figure 2, and the overall early hepatorenal stress response across phenotype-defined groups is presented in Figure 3.

### 3.3. Multivariable and Longitudinal Determinants of Perioperative MELD Response

In multivariable linear regression analyses, both female sex and diabetes mellitus remained correlated with lower postoperative MELD values. Among female patients, we observed a significant association with a reduction in postoperative MELD (β = −2.54, *p* < 0.001), whereas diabetes mellitus was associated with a smaller postoperative MELD increase (β = −1.07, *p* = 0.028). Baseline MELD remained a strong predictor of absolute postoperative MELD level (β = 0.80, *p* < 0.001), but did not significantly predict the magnitude of perioperative MELD change. No significant interaction between sex and diabetes mellitus was identified, indicating additive rather than synergistic effects.

Longitudinal analysis using generalized estimating equations confirmed a significant perioperative rise in MELD on postoperative day 1, followed by partial recovery by postoperative day 6 (*p* < 0.001). Importantly, both female sex and diabetes mellitus were associated with attenuation of this trajectory, supporting the interpretation that the observed differences reflect distinct response patterns over time rather than isolated intergroup differences at individual time points.

To identify determinants of an exaggerated perioperative response, patients were additionally categorized according to the presence or absence of a high-surge MELD phenotype. In logistic regression analysis, female sex (OR 0.09, *p* = 0.024) and diabetes mellitus (OR 0.29, *p* = 0.024) were independently associated with a substantially lower likelihood of developing this phenotype. Baseline MELD was not predictive of high-surge status.

### 3.4. Clinical Correlates of the High-Surge MELD Phenotype

Clinical characteristics of patients with high- and low-surge MELD phenotypes are summarized in Table 3. The two analyzed populations did not show significant differences in age, sex distribution, body mass index, cardiovascular comorbidities, smoking status, or the extent of coronary artery disease. Baseline MELD values were likewise comparable between groups.

As expected, patients with the high-surge phenotype exhibited significantly higher MELD values on postoperative day 1 and markedly greater ΔMELD_01_ values than those with the low-surge phenotype, whereas MELD_6_ and ΔMELD_06_ remained similar between groups. Most importantly, the high-surge phenotype was associated with prolonged recovery. Patients with high MELD scores had significantly longer overall hospitalization than those with lower MELD scores (14.8 ± 2.1 vs. 9.2 ± 1.2 days, *p* < 0.001), as well as longer postoperative hospitalization (9.7 ± 2.0 vs. 6.5 ± 1.0 days, *p* < 0.001). In contrast, intensive care unit stay did not differ significantly between groups. These findings indicate that an exaggerated early postoperative MELD response identifies patients with slower postoperative recovery despite otherwise broadly comparable baseline clinical characteristics.

## 4. Discussion

The present study shows that perioperative MELD trajectories may reflect a dynamic hepatorenal stress response after surgical coronary revascularization and that this response is shaped by both sex and metabolic status. In contrast to baseline MELD values, which primarily reflect preoperative organ function, the perioperative rise and recovery of MELD appear to capture the organism’s response to surgical stress in real time. This distinction appears important because our results suggest that not only the absolute MELD value but also the magnitude of its perioperative change may identify distinct physiological response patterns with potential clinical relevance.

Although MELD was originally developed for patients with advanced liver disease, its components—bilirubin, creatinine, and INR—reflect processes that are highly relevant in the perioperative setting. The pronounced increase observed on postoperative day 1 most likely represents the combined impact of systemic inflammation, transient hepatic hypoperfusion, renal dysfunction, and coagulation disturbances induced by surgery. Notably, baseline MELD did not predict the magnitude of this early postoperative response, suggesting that dynamic MELD assessment may provide information not captured by static preoperative measurements alone. In this context, MELD should be viewed less as a marker of pre-existing liver dysfunction and more as an integrated indicator of acute multiorgan stress in the postoperative period [14,15].

An additional factor that may have contributed to the observed persistence of elevated INR values on postoperative day 6 is the use of enoxaparin for routine thromboprophylaxis. Previous studies have demonstrated that postoperative enoxaparin administration may be associated with mild increases in INR, even in the absence of intrinsic hepatic dysfunction [16]. Therefore, the sustained INR elevation observed in our cohort should be interpreted cautiously, as it may partly reflect pharmacological effects rather than ongoing impairment of hepatic synthetic function.

One of the most consistent findings of the present study was the attenuated MELD response observed in women [17,18]. Although women also had lower baseline MELD values, multivariable analysis confirmed that female sex remained independently associated with lower postoperative MELD values and a smaller early postoperative increase, even after adjustment for baseline levels. This suggests that the observed difference is not simply a reflection of lower preoperative values, but rather points to a genuine difference in physiological stress responsiveness. Such an interpretation is consistent with earlier reports showing sex-related variation in inflammatory activation and vascular reactivity in cardiac surgical populations, including observations from our own group [19,20]. Potential biological explanations include differences in endothelial function, inflammatory signaling, and mitochondrial resilience. Previous experimental and clinical work suggests that women may exhibit lower inflammatory activation and better-preserved microvascular function during acute stress, which could translate into a less pronounced hepatorenal response after surgery [21,22]. Although these mechanisms cannot be directly verified in the present study, the overall pattern is in line with broader evidence indicating sex-related differences in cardiovascular and systemic stress responses [23,24,25].

Sex-related differences in cardiovascular disease extend beyond the perioperative setting and have been widely documented across a range of conditions, including coronary artery disease, heart failure, and vascular dysfunction [25,26,27,28]. Women often exhibit distinct clinical presentations, differences in microvascular function, and variations in inflammatory and hormonal responses compared with men [29,30]. Estrogen-related effects on endothelial function, nitric oxide bioavailability, and mitochondrial efficiency have been proposed as key mechanisms contributing to these differences [31,32]. In the context of cardiac surgery, sex-based variation may reflect differences in vascular reactivity, oxidative stress response, and immune activation [33,34,35]. Experimental data suggest that women may demonstrate reduced inflammatory cytokine release and improved microcirculatory resilience during acute stress, which could attenuate secondary organ dysfunction, including hepatorenal impairment [36]. Additionally, differences in body composition, pharmacokinetics, and coagulation pathways may further contribute to the observed variability in perioperative MELD trajectories [37,38,39]. Although the present study was not designed to directly investigate mechanistic pathways, the observed sex-related differences in MELD dynamics are consistent with a broader body of evidence indicating that biological sex is an important modifier of systemic stress responses and should be considered in perioperative risk assessment.

The association between diabetes mellitus and a lower MELD score was more surprising and should be interpreted carefully. Diabetes is usually linked with worse cardiovascular outcomes, so the attenuated perioperative MELD rise observed here should not be taken as evidence of lower overall risk. Rather, this finding may reflect altered physiological adaptation. One possible explanation is chronic metabolic reprogramming in diabetes, including changes in substrate utilization, cellular preconditioning, and inflammatory signaling, which could blunt the immediate biochemical response to acute ischemic or inflammatory stress [40,41,42]. Another possibility is that modern antidiabetic treatment, especially sodium-glucose cotransporter 2 inhibitors, may have influenced perioperative physiology. At this stage, however, these explanations remain speculative, and the observed association should be regarded as hypothesis-generating rather than definitive.

An important aspect of the present analysis is the identification of a high-surge MELD phenotype. This concept offers a simple way to describe patients with an exaggerated early postoperative hepatorenal response. Unlike conventional risk models, which depend largely on static preoperative variables, this approach focuses on the dynamics of physiological adaptation. In our cohort, the distribution of this phenotype followed a clear gradient across sex and diabetes strata, supporting the idea that biological and metabolic factors modulate the intensity of the perioperative stress response. At the same time, because the present study did not include major postoperative endpoints such as acute kidney injury, hepatic dysfunction, or mortality, the high-surge phenotype should currently be interpreted as a marker of physiological response rather than as a direct surrogate of clinical risk.

Even so, its association with prolonged hospitalization makes it clinically interesting. Patients with exaggerated MELD elevation on postoperative day 1 required longer overall and postoperative in-hospital recovery despite otherwise similar baseline demographic and clinical characteristics. No single complication pattern explained this difference, but prolonged recovery itself may reflect increased physiological vulnerability. From a practical standpoint, these findings suggest that perioperative MELD monitoring could help identify patients with an intensified stress response who may benefit from closer surveillance or more individualized postoperative management. More broadly, our results support the growing concept that perioperative risk stratification should move beyond static baseline scores and incorporate biomarker trajectories that better reflect acute physiological adaptation [43,44].

There are several limitations associated with the interpretation of these findings. First, this was a single-center study with a relatively small sample size, which limits generalizability. Second, the retrospective design introduces the possibility of residual confounding despite multivariable adjustment. Third, the study was restricted to patients undergoing elective off-pump coronary artery bypass grafting, so the findings may not be directly transferable to other cardiac surgical populations. In addition, major postoperative clinical outcomes were not systematically analyzed, so the identified MELD trajectories cannot yet be directly linked to hard endpoints. The predefined laboratory time points may also not have fully captured the true peak of postoperative organ dysfunction. Finally, the high-surge phenotype was defined using a cohort-specific upper quartile threshold and should therefore be considered exploratory.

Taken together, the findings of this study suggest that perioperative MELD trajectories provide clinically meaningful information about the acute hepatorenal response to surgical stress. This response appears to be modulated by sex and diabetes status, and an exaggerated early MELD surge is associated with delayed postoperative recovery. Dynamic MELD assessment may therefore complement conventional perioperative risk models by capturing a dimension of physiological vulnerability that static preoperative variables do not reflect. Further studies are needed to determine whether these trajectory-based phenotypes are associated with postoperative complications, organ dysfunction, and longer-term outcomes.

## 5. Conclusions

Perioperative changes in MELD appear to reflect a dynamic hepatorenal response to cardiac surgery rather than baseline organ status alone. In the present study, this response was not uniform but varied by sex and diabetes status, with women and patients with diabetes showing a less pronounced postoperative MELD rise. These findings support the presence of distinct physiological response patterns that may not be captured by conventional preoperative risk assessment.

Importantly, patients with a high-surge MELD phenotype had significantly longer hospitalization despite otherwise comparable baseline characteristics. This suggests that dynamic MELD assessment may identify clinically relevant physiological vulnerability and provide information beyond that offered by static preoperative measures.

Taken together, our findings support the potential value of trajectory-based biomarkers in perioperative evaluation. Our results support the importance of incorporating sex-specific physiological differences into perioperative risk stratification models.

Further studies are needed to determine whether perioperative MELD trajectories are associated with specific postoperative complications, organ dysfunction, and longer-term clinical outcomes.

## Figures and Tables

**Table 1:** Characteristics of the study cohort stratified by sex and diabetes status.

Parameter	Whole Group *n* = 111	Females (F) *n* = 24	Men (M) *n* = 87	*p* (M vs. F)	No Diabetes *n* = 64	Diabetes Mellitus *n* = 47	*p* (no vs. DM)
**Demographic characteristics**							
Age, years, median (Q1–Q3)	68 (63–72)	72 (67–74)	68 (62–72)	0.037	69 (63–73)	67 (64–72)	0.919
Male sex, *n* (%)	87 (78.4)	0 (0)	87 (100.0)	<0.001	49 (76.6)	38 (80.9)	0.592
BMI, kg/m^2^, median (Q1–Q3)	29 (25–32)	27 (24–29)	30 (27–32)	0.016	29 (24–31)	29 (27–32)	0.263
BMI > 30 kg/m^2^, *n* (%)	28 (25.2)	10 (41.7)	14 (16.1)	0.055	14 (21.9)	14 (29.8)	0.355
**Comorbidities**							
Arterial hypertension, *n* (%)	104 (93.7)	21 (87.5)	83 (95.4)	0.163	59 (92.2)	45 (95.7)	0.452
Diabetes mellitus, *n* (%)	47 (42.3)	9 (37.5)	38 (43.7)	0.592	0 (0)	47 (100)	<0.001
Dyslipidemia, *n* (%)	57 (51.4)	8 (33.3)	47 (54.0)	0.048	34 (53.1)	23 (48.9)	0.667
PAD, *n* (%)	22 (19.8)	11 (45.8)	11 (12.6)	<0.001	11 (17.2)	11 (23.4)	0.421
Prior stroke, *n* (%)	7 (6.3)	2 (8.3)	5 (5.7)	0.652	6 (9.4)	1 (2.1)	0.124
Active smoking, *n* (%)	24 (21.6)	8 (33.3)	16 (18.4)	0.118	14 (21.9)	10 (21.3)	0.943
**Coronary artery disease**							
Number of involved arteries, mean (SD)	1.9 (1.2)	1.5 (1.3)	2.0 (1.2)	0.484	2.0 (1.2)	2.0 (1.2)	0.690
Left main disease, *n* (%)	46 (41.4)	11 (45.8)	35 (40.2)	0.488	28 (43.8)	18 (38.3)	0.808
LAD disease, *n* (%)	90 (81.1)	18 (75.0)	72 (82.8)	0.395	49 (76.6)	41 (87.2)	0.159
Cx disease, *n* (%)	60 (54.1)	14 (58.3)	46 (52.9)	0.639	36 (56.3)	24 (51.1)	0.592
RCA disease, *n* (%)	67 (60.4)	14 (58.3)	53 (60.9)	0.823	40 (62.5)	27 (57.4)	0.595
**Preoperative echocardiography**							
LVD, mm, median (Q1–Q3)	46 (41–51)	42 (40–49)	46 (42–51)	0.143	46 (42–49)	47 (41–52)	0.875
RVD, mm, median (Q1–Q3)	28 (26–31)	25 (21–27)	29 (27–32)	<0.001	28 (27–31)	28 (26–32)	0.897
IVS, mm, median (Q1–Q3)	13 (11–15)	11 (10–14)	13 (11–15)	0.013	13 (11–14)	12 (11–14)	0.293
EF, %, median (Q1–Q3)	55 (50–60)	55 (50–60)	59 (50–63)	0.509	55 (50–60)	60 (50–60)	0.696
**Postoperative echocardiography**							
LVED, cm, median (Q1–Q3)	46 (41–49)	42 (38–48)	46 (42–50)	0.086	45 (41–49)	47 (42–52)	0.131
RVD, cm, median (Q1–Q3)	28 (25–30)	24 (21–26)	29 (26–31)	<0.001	28 (25–31)	28 (24–30)	0.666
IVS, cm, median (Q1–Q3)	12 (11–14)	11 (9–13)	13 (11–15)	0.006	13 (11–15)	12 (11–13)	0.080
LVEF, %, median (Q1–Q3)	60 (50–60)	55 (50–60)	60 (50–60)	0.624	59 (50–60)	60 (55–60)	0.388

**Abbreviations:** BMI, body mass index; Cx, circumflex artery; IVS, interventricular septal thickness; LAD, left anterior descending artery; LVD, left ventricular diameter; EF, ejection fraction of left ventricle; PAD, peripheral artery disease; Q, quartile; RCA, right coronary artery; RVD, right ventricular end-diastolic diameter.

**Table 2:** Perioperative laboratory parameters according to sex–diabetes phenotype.

Parameter	Men Without Diabetes (1) *n* = 49	Men with Diabetes (2) *n* = 38	Women Without Diabetes (3) *n* = 15	Women with Diabetes (4) *n* = 9	*p* 1 vs. 2	*p* 1 vs. 3	*p* 1 vs. 4	*p* 2 vs. 3	*p* 2 vs. 4	*p* 2 vs. 3
**Preoperative**										
WBC, 10 × 9/L, median (Q1–Q3)	6.8 (5.9–8.6)	7.5 (6.4–9.4)	7.4 (6.9–9.2)	9.8 (6.6–10.1)	0.181	0.103	0.071	0.694	0.306	0.721
Hemoglobin, mmol/L, median (Q1–Q3)	9.0 (8.3–9.4)	8.8 (8.0–9.3)	8.7 (8.1–9.0)	8.3 (8.1–9.0)	0.613	0.088	0.060	0.229	0.217	0.858
Platelets, 10 × 9/L, median (Q1–Q3)	206 (171–250)	214 (174–247)	249 (217–306)	236 (184–273)	0.824	0.016	0.308	0.012	0.234	0.438
ALT, IU/L, median (Q1–Q3)	29 (23–40)	35 (27–57)	28 (19–31)	25 (21–32)	0.115	0.093	0.237	0.012	0.096	1.000
AST, IU/L, median (Q1–Q3)	25 (20–33)	28 (24–35)	23 (20–26)	22 (16–36)	0.276	0.227	0.382	0.030	0.223	0.698
Bibilirubin total, mg/dL, median (Q1–Q3)	0.62 (0.40–0.79)	0.50 (0.42–0.62)	0.46 (0.39–0.72)	0.46 (0.39–0.71)	0.187	0.541	0.465	0.968	0.808	1.000
Total Cholesterol, mmol/L, median (Q1–Q3)	3.46 (3.00–4.04)	3.68 (2.80–4.77)	3.72 (3.27–4.81)	3.56 (3.17–4.18)	0.397	0.192	0.632	0.694	0.903	0.548
Serum Creatinine, mg/dL, median (Q1–Q3)	0.95 (0.84–1.08)	1.00 (0.91–1.19)	0.88 (0.70–1.00)	0.94 (0.79–1.09)	0.130	0.094	0.813	0.012	0.285	0.200
INR, median (Q1–Q3)	1.06 (1.04–1.11)	1.04 (0.99–1.06)	1.03 (1.03–1.08)	0.97 (0.94–0.99)	0.018	0.305	<0.001	0.416	0.017	0.004
MELD-0, median (Q1–Q3)	5.76 (5.20–6.67)	5.98 (5.05–6.92)	5.26 (5.25–5.78)	4.81 (4.56–5.54)	0.786	0.369	0.033	0.356	0.033	0.098
Troponin—I, ng/L, median (Q1–Q3)	0.01 (0.02–0.04)	0.01 (0.01–0.03)	0.01 (0.00–0.02)	0.01 (0.00–0.03)	0.274	0.047	0.393	0.218	0.701	0.875
**1st postoperative day**										
WBC-1, 10 × 9/L, median (Q1–Q3)	11.7 (9.3–14.0)	10.7 (9.9–13.3)	12.5 (10.6–14.9)	12.8 (10.6–13.9)	0.650	0.296	0.660	0.130	0.332	0.766
Hemoglobin-1, mmol/L, median (Q1–Q3)	7.3 (6.9–7.8)	7.4 (6.7–7.7)	7.2 (6.9–7.5)	7.0 (6.4–7.2)	0.844	0.441	0.091	0.537	0.141	0.295
ALT-1, IU/L, median (Q1–Q3)	28 (19–41)	32 (23–44)	23 (16–34)	24 (20–27)	0.489	0.195	0.178	0.080	0.045	0.682
AST-1, IU/L, median (Q1–Q3)	34 (26–44)	34 (29–46)	27 (23–35)	32 (28–39)	0.960	0.136	0.806	0.095	0.648	0.308
Bibilirubin total-1, mg/dL, median (Q1–Q3)	0.89 (0.71–1.06)	0.76 (0.54–1.06)	0.70 (0.52–0.88)	0.50 (0.39–0.51)	0.080	0.023	<0.001	0.465	0.025	0.161
Creatinine-1, mg/dL, median (Q1–Q3)	1.27 (1.18–1.62)	1.43 (1.24–1.66)	1.2 (1.1–1.3)	1.2 (1.1–1.3)	0.061	0.196	0.221	0.004	0.014	1.000
INR-1, median (Q1–Q3)	1.19 (1.14–1.27)	1.17 (1.12–1.20)	1.14 (1.11–1.22)	1.15 (1.05–1.16)	0.037	0.060	0.005	0.514	0.074	0.256
MELD-1, median (Q1–Q3)	17.3 (15.8–18.1)	16.9 (15.1–18.4)	14.03 (13.13–15.13)	13.15 (11.81–14.85)	0.353	0.024	0.020	0.002	0.003	0.512
Troponin—I, ng/L, median (Q1–Q3)	1.27 (0.55–2.30)	1.01 (0.56–1.62)	0.56 (0.34–0.92)	0.40 (0.27–1.98)	0.246	0.017	0.229	0.079	0.740	1.000
**6th postoperative day**										
WBC-6, 10 × 9/L, median (Q1–Q3)	6.8 (5.3–8.0)	6.9 (5.9–8.4)	8.0 (6.3–8.9)	7.7 (6.1–8.0)	0.653	0.103	0.606	0.289	0.761	0.438
Hemoglobin-6, mmol/L, median (Q1–Q3)	6.6 (6.4–7.0)	6.6 (6.2–6.9)	6.8 (6.7–7.5)	7.3 (6.8–7.3)	0.131	0.098	0.016	0.016	0.003	0.471
ALT-6, IU/L, median (Q1–Q3)	15 (11–23)	14 (9–19)	18 (11–30)	15 (12–19)	0.681	0.711	1.000	0.422	1.000	1.000
AST-6, IU/L, median (Q1–Q3)	23 (20–32)	20 (15–37)	40 (25–58)	37 (31–41)	0.328	0.114	0.417	0.070	0.596	1.000
Bibilirubin total-6, mg/dL, median (Q1–Q3)	0.57 (0.055–0.69)	0.28 (0.26–0.49)	0.36 (0.25–0.47)	0.48 (0.33–0.56)	0.008	0.151	0.400	0.350	1.000	1.000
Creatinine-6, mg/dL, median (Q1–Q3)	0.81 (0.70–1.06)	0.89 (0.72–1.12)	0.65 (0.50–0.75)	0.84 (0.59–0.95)	0.291	0.003	0.373	<0.001	0.142	0.196
INR-6, median (Q1–Q3)	1.11 (1.08–1.18)	1.14 (1.07–1.18)	1.12 (1.04–1.18)	0.98 (0.97–1.00)	0.827	0.794	0.007	0.823	0.067	0.183
MELD-6, median (Q1–Q3)	5.78 (4.93–6.23)	5.45 (5.21–7.03)	5.54 (3.77–5.76)	4.22 (3.91–5.19)	0.508	0.926	0.131	0.518	0.200	0.333
Troponin—I-6, ng/L, median (Q1–Q3)	0.21 (0.08–1.30)	0.38 (0.22–0.56)	0.19 (0.06–1.45)	0.23 (0.09–1.44)	0.942	0.945	1.000	0.966	0.927	0.889

**Abbreviations:** ALT, alanine aminotransferase; AST, aspartate aminotransferase; DM, diabetes mellitus; INR, international normalized ratio; MELD, Model for End-Stage Liver Disease; Q, quartile; WBC, white blood cell count.

**Table 3:** Baseline characteristics, perioperative MELD parameters, and hospitalization outcomes according to high-surge MELD phenotype.

Variable	High MELD Surge (*n* = 28)	Low MELD Surge (*n* = 83)	*p*-Value
**Demographic characteristics**			
Age, y, (median (Q1–Q3))	68.0 (63.0–72.0)	68.0 (63.0–72.0)	0.991
Male sex, *n* (%)	22 (78.6)	65 (78.3)	1.000
BMI, kg/m^2^	29.1 (25.6–32.5)	28.9 (25.1–31.8)	0.842
BMI > 30 kg/m^2^, *n* (%)	7 (25.0)	21 (25.3)	1.000
**Comorbidities**			
Hypertension, *n* (%)	26 (92.9)	78 (94.0)	1.000
Diabetes mellitus, *n* (%)	11 (39.3)	36 (43.4)	0.826
Dyslipidemia, *n* (%)	14 (50.0)	43 (51.8)	1.000
PAD, *n* (%)	6 (21.4)	16 (19.3)	0.794
Prior stroke, *n* (%)	2 (7.1)	5 (6.0)	1.000
Active smoking, *n* (%)	6 (21.4)	18 (21.7)	1.000
**Coronary artery disease**			
LMCA disease, *n* (%)	11 (39.3)	35 (42.2)	0.826
LAD disease, *n* (%)	23 (82.1)	67 (80.7)	1.000
Cx disease, *n* (%)	15 (53.6)	45 (54.2)	1.000
RCA disease, *n* (%)	17 (60.7)	50 (60.2)	1.000
**MELD parameters** median (Q1–Q3)			
MELD_0_	5.8 (5.2–6.7)	5.8 (5.2–6.7)	0.964
MELD_1_	17.8 (16.9–19.2)	15.9 (14.8–17.2)	<0.001
MELD_6_	5.9 (4.8–6.4)	5.6 (4.7–6.2)	0.412
ΔMELD_01_	12.1 (11.3–13.9)	8.9 (7.8–10.1)	<0.001
ΔMELD_06_	0.3 (−0.9–1.6)	0.2 (−1.0–1.3)	0.721
**Clinical outcomes**			
Overall hospitalization, days, mean (SD)	12.8 ± 2.1	9.2 ± 1.2	<0.001
Intensive care unit stay, h	28 (5)	24 (4)	0.285
Postoperative hospitalization, days, mean (SD)	9.7 ± 2.0	6.5 ± 1.0	<0.001

**Abbreviations:** BMI, body mass index; Cx, circumflex artery; LAD, left anterior descending artery; LMCA, left main coronary artery; MELD, Model for End-Stage Liver Disease; PAD, peripheral artery disease; Q, quartile; RCA, right coronary artery; y—years.

## Data Availability

The data presented in this study are available on request from the corresponding author.

## Abbreviations

The following is a list of abbreviations applied in this manuscript:

ALT	alanine aminotransferase
AST	aspartate aminotransferase
BMI	body mass index
CABG	coronary artery bypass grafting
Cx	circumflex artery
DM	diabetes mellitus
GEE	generalized estimating equations
ICU	intensive care unit
INR	international normalized ratio
IVS	interventricular septal thickness
LAD	left anterior descending artery
LDL	low-density lipoprotein
LMCA	left main coronary artery
LVED	left ventricular end-diastolic diameter
LVEF	left ventricular ejection fraction
MELD	Model for End-Stage Liver Disease
OPCAB	off-pump coronary artery bypass grafting
PAD	peripheral artery disease
POD	postoperative day
Q	quartile
RCA	right coronary artery
RVD	right ventricular end-diastolic diameter
SD	standard deviation
SGLT2	sodium-glucose cotransporter 2
WBC	white blood cell count

## References

[1] Ruf A., Dirchwolf M., Freeman R.B. (2022). From Child-Pugh to MELD score and beyond: Taking a walk down memory lane. Ann. Hepatol..

[2] Pathare P., Elbayoumi M., Weyand M., Griesbach C., Haig F. (2023). MELD-score for risk stratification in cardiac surgery. Heart Vessel..

[3] Yu X., Zhou R., Tan W., Wang X., Zheng X., Huang Y., Chen J., Li B., Liu X., Li Z. (2024). Evidence-based incorporation of key parameters into MELD score for acute-on-chronic liver failure. eGastroenterology.

[4] Urbanowicz T.K., Olasińska-Wiśniewska A., Michalak A., Gąsecka A., Rodzki M., Perek B., Jemielity M. (2021). Cardioprotective effect of low level of LDL cholesterol on perioperative myocardial injury in off-pump coronary artery bypass grafting. Medicina.

[5] Błażejowska E., Urbanowicz T., Gąsecka A., Olasińska-Wiśniewska A., Jaguszewski M.J., Targoński R., Szarpak Ł., Filipiak K.J., Perek B., Jemielity M. (2021). Diagnostic and prognostic value of miRNAs after coronary artery bypass grafting: A review. Biology.

[6] Urbanowicz T., Michalak M., Al-Imam A., Olasińska-Wiśniewska A., Rodzki M., Witkowska A., Haneya A., Buczkowski P., Perek B., Jemielity M. (2022). The significance of systemic immune-inflammation index for mortality prediction in diabetic patients treated with off-pump coronary artery bypass surgery. Diagnostics.

[7] Urbanowicz T., Michalak M., Gąsecka A., Perek B., Rodzki M., Bociański M., Straburzyńska-Migaj E., Jemielity M. (2021). Postoperative neutrophil to lymphocyte ratio as an overall mortality midterm prognostic factor following OPCAB. Clin. Pract..

[8] Gourier S., Léon K., Vermeersch V., Bouras M., Langeron O., Caillard A. (2025). Sex-based differences in anesthesia approaches and outcomes: A narrative review. Anaesth. Crit. Care Pain Med..

[9] Walters S.M., Richter E.W., Lutzker T., Patel S., Vincent A.N., Kleiman A.M. (2020). Perioperative considerations regarding sex in solid organ transplantation. Anesthesiol. Clin..

[10] Nerenberg K.A., Roeters van Lennep J.E. (2021). Advancing sex and gender considerations in perioperative cardiovascular-risk assessment. Can. J. Cardiol..

[11] Zheng Q., Zhou J., Zhang Y., Wang T., Wu D., Pu Q., Mei J., Liao H., Liu L. (2024). Insights into sex differences in perioperative outcomes of non-small cell lung cancer patients. Transl. Lung Cancer Res..

[12] Di Florio D.N., Sin J., Coronado M.J., Atwal P.S., Fairweather D. (2020). Sex differences in inflammation, redox biology, mitochondria and autoimmunity. Redox Biol..

[13] Kirkman D.L., Robinson A.T., Rossman M.J., Seals D.R., Edwards D.G. (2021). Mitochondrial contributions to vascular endothelial dysfunction, arterial stiffness, and cardiovascular diseases. Am. J. Physiol. Heart Circ. Physiol..

[14] Szyguła-Jurkiewicz B., Szczurek-Wasilewicz W., Gąsior M., Copik I., Małyszek-Tumidajewicz J., Skrzypek M., Romuk E., Zembala M., Zembala M., Przybyłowski P. (2021). Oxidative stress markers and modified model for end-stage liver disease are associated with outcomes in patients with advanced heart failure receiving bridge therapy with continuous-flow left ventricular assist devices. Antioxidants.

[15] Kozluca V., Akbulut I.M., Tan T.S., Gulyigit H., Ozerdem M.E., Sayin T. (2025). Diuretic response prediction with MELD score in heart failure. Clin. Cardiol..

[16] Kelecy M.W., Shutt T., Rostas J., Martin R.C.G. (2018). Clinical effect of enoxaparin on international normalized ratio following hepato-pancreatico-biliary and gastroesophageal resection. J. Surg. Oncol..

[17] Allen A.M., Heimbach J.K., Larson J.J., Mara K.C., Kim W.R., Kamath P.S., Therneau T.M. (2018). Reduced access to liver transplantation in women: Role of height, MELD exception scores, and renal function underestimation. Transplantation.

[18] Bittermann T., Mahmud N., Weinberg E.M., Reddy K.R. (2023). MELD 3.0 leads to heterogeneous prioritization of men and women on the liver transplant waiting list. Liver Transpl..

[19] Urbanowicz T., Michalak M., Olasińska-Wiśniewska A., Haneya A., Straburzyńska-Migaj E., Bociański M., Jemielity M. (2021). Gender differences in coronary artery diameters and survival results after off-pump coronary artery bypass (OPCAB) procedures. J. Thorac. Dis..

[20] Urbanowicz T.K., Michalak M., Olasińska-Wiśniewska A., Żukowski M., Koczorowski K., Łasowski B., Woźnicki M., Filipiak K.J., Tykarski A., Jemielity M. (2024). Left main coronary artery disease treated with beating heart surgery: 10-year single center results. Postepy Kardiol. Interwencyjnej.

[21] Greaney J.L., Saunders E.F.H., Santhanam L., Alexander L.M. (2019). Oxidative stress contributes to microvascular endothelial dysfunction in men and women with major depressive disorder. Circ. Res..

[22] Fonkoue I.T., Michopoulos V., Park J. (2020). Sex differences in post-traumatic stress disorder risk: Autonomic control and inflammation. Clin. Auton. Res..

[23] Tower J., Pomatto L.C.D., Davies K.J.A. (2020). Sex differences in the response to oxidative and proteolytic stress. Redox Biol..

[24] Wang-Heaton H., Wingard M.C., Dalal S., Shook P.L., Connelly B.A., Johnson P., Nichols P.L., Singh M., Singh K. (2024). ATM deficiency differentially affects expression of proteins related to fatty acid oxidation and oxidative stress in a sex-specific manner in response to Western-type diet prior to and following myocardial infarction. Life Sci..

[25] Cristallo M., Furci F., Cascario M., Gangemi S., Nettis E. (2025). Sex differences in oxidative stress concerning allergic diseases. Biomolecules.

[26] Ciutac A.M., Pana T., Dawson D., Myint P.K. (2025). Sex-related differences in heart failure patients: Physiological mechanisms of cardiovascular ageing and evidence-based sex-specific medical therapies. Ther. Adv. Cardiovasc. Dis..

[27] Lüscher T., Flammer A., Kuster G. (2023). Sex and Cardiovascular Disease. Cardiovasc. Med..

[28] Alamat L., Rana A., Lopez Candales A., Sawalha K., Shaw-Devine A. (2025). True Impact of Sex Differences in Diagnosis and Management of Cardiovascular Disease. Cureus.

[29] Gupta A., Gupta A.K. (2026). Small vessels, big impact: Understanding women’s microvascular heart disease. Curr. Opin. Physiol..

[30] Ivanova M.M., Dao J., Friedman A., Kasaci N., Goker-Alpan O. (2025). Sex Differences in Circulating Inflammatory, Immune, and Tissue Growth Markers Associated with Fabry Disease-Related Cardiomyopathy. Cells.

[31] SenthilKumar G., Katunaric B., Bordas-Murphy H., Sarvaideo J., Freed J.K. (2023). Estrogen and the Vascular Endothelium: The Unanswered Questions. Endocrinology.

[32] Xiang D., Liu Y., Zhou S., Zhou E., Wang Y. (2021). Protective Effects of Estrogen on Cardiovascular Disease Mediated by Oxidative Stress. Oxid Med. Cell Longev..

[33] Squiccimarro E., Lorusso R., Margari V., Labriola C., Whitlock R., Paparella D. (2025). Sex-related differences in systemic inflammatory response and outcomes after cardiac surgery and cardiopulmonary bypass. Interdiscip Cardiovasc. Thorac. Surg..

[34] Chong-Nguyen C., Fuentes-Artiles R., Pilgrim T., Yilmaz B., Döring Y. (2026). The gut-heart axis in coronary artery disease: A scoping and narrative review of sex-based microbial and metabolic disparities. Biol. Sex Differ..

[35] Kuhn L., Schupp T., Steinke P., Weidner K., Bertsch T., Rusnak J., Jannesari M., Siegel F., Duerschmied D., Behnes M. (2025). Sex-Based Differences and Outcomes in Unselected Patients Undergoing Coronary Angiography. J. Clin. Med..

[36] Balcerowska M., Kwaśnik P. (2025). The multifaceted impact of stress on immune function. Mol. Biol. Rep..

[37] Hamaguchi Y., Kaido T., Okumura S., Kobayashi A., Shirai H., Yao S., Yagi S., Kamo N., Uemoto S. (2020). Including body composition in MELD scores improves mortality prediction among patients awaiting liver transplantation. Clin. Nutr..

[38] Kim Y.K., Shin W.J., Song J.G., Jun I.G., Kim H.Y., Seong S.H., Sang B.H., Hwang G.S. (2010). Factors associated with changes in coagulation profiles after living donor hepatectomy. Transpl. Proc..

[39] McChord J., Ong P. (2024). Bridging the Gender Gap in Cardiovascular Medicine: Addressing Drug Intolerances and Personalized Care for Women with Angina/Ischemia with Non-Obstructive Coronary Artery Disease. J. Cardiovasc. Dev. Dis..

[40] Varga S., Juhász L., Gál P., Bogáts G., Boros M., Palásthy Z., Szabó A., Kaszaki J. (2019). Neuronal nitric oxide mediates the anti-inflammatory effects of intestinal ischemic preconditioning. J. Surg. Res..

[41] Raevens S., Boert M., Fallon M.B. (2022). Hepatopulmonary syndrome. JHEP Rep..

[42] Wahlström K.L., Hansen H.F., Kvist M., Burchardt J., Lykkesfeldt J., Gögenur I., Ekeloef S. (2023). Effect of remote ischemic preconditioning on perioperative endothelial dysfunction in non-cardiac surgery: A randomised clinical trial. Cells.

[43] Jaffer A., Yang K., Ebrahim A., Brown A.N., El-Andari R., Dokollari A., Gregory A.J., Adams C., Kent W.D.T., Fatehi Hassanabad A. (2025). Optimizing recovery in cardiac surgery: A narrative review of enhanced recovery after surgery protocols and clinical outcomes. Med. Sci..

[44] Azari A., Baradaran Rahimi V., Moravvej Z., Rahsepar A.A., Ghayour-Mobarhan M., Salehi M., Bigdelu L. (2023). Antioxidant activity in off and on-pump coronary artery bypass grafting and valve replacement surgery. J. Basic Clin. Physiol. Pharmacol..

